# The Bay Area Muslim mental health community advisory board: evaluation of a community based participatory approach

**DOI:** 10.1017/S2045796022000786

**Published:** 2023-01-31

**Authors:** S. S. Ali, I. Mahoui, R. Hassoun, H. Mojaddidi, R. Awaad

**Affiliations:** 1 Department of Psychiatry and Behavioral Sciences, Stanford University School of Medicine, Stanford, USA; 2 Stanford Muslims Mental Health and Wellbeing Program, Stanford University School of Medicine, Stanford, USA; 3 School of Public Health, George Washington University, Washington, USA; 4 Community Advisory Board Member, Muslim Community Association, Santa Clara, USA; 5 Stanford Muslim Mental Health and Islamic Psychology Lab, Department of Psychiatry and Behavioral Sciences, Stanford University School of Medicine, Stanford, USA

**Keywords:** Community mental health, minority issues and cross cultural psychiatry, multicultural, religion, research design and methods

## Abstract

**Aims:**

The aim of this paper is to present a novel case for the formation, operation and evaluation of a community advisory aboard comprised of Muslims residing in the San Francisco Bay Area, California that utilised a community based participatory approach to address local Muslim mental health needs. The CAB was recruited in partnership with the Muslim Community Association (MCA), one of the largest Islamic centres in the San Franscisco Bay Area. In addition to describing the development of the CAB, the authors present the findings of the evaluation and synthesis of best processes based on CAB members' feedback.

**Methods:**

To evaluate the perceived community advisory board members' perceptions of their roles and elicit feedback on how to enhance the relationship between the university team and the CAB, an evaluation was conducted by an independent team who was not part of the research process. Data was collected using anonymous individual surveys and small group open discussions that were conducted over three evaluation meetings. The evaluation utilised mixed method data collection strategies using questions from Schulz *et al.* (2003, Evaluation and Program Planning 26, 249–262), an instrument for evaluating dimensions of group dynamics within CBPR partnerships.

**Results:**

Results of the evaluation within the sphere of CAB operation indicated that CAB members found the greatest satisfaction from their contributions through direct participation in the research activities that were conducted by the university-CAB team. The collective responses indicated that most CAB members were satisfied with trust built between the university-CAB team and the diversity represented in the members of the board. However, given that the Bay Area is home to a very diverse Muslim community, challenges in recruiting representatives that account for all possible self-identifying groups was reported by the CAB with recommendations to recruit religious leaders. Recommendations also included eliciting funds for potential financial compensation for CAB members.

**Conclusions:**

The Stanford-San Francisco Bay Area CAB demonstrated that empowering community members through direct participation, creating channels and safe spaces for feedback help create community rooted research that carry the true voices of marginalised communities and reflects their evolving needs

## Introduction

Over the last two decades there has been a growing interest in research aimed at addressing the mental health needs of faith-based communities and their conceptualisation of mental health and wellbeing (Cinnirella and Loewenthal, 1999; Aloud and Rathur, 2009; Sullivan *et al*., 2014). This includes a growing population of Muslim Americans who currently comprise 1.1% of the American population (Pew Research, 2011). It has been well documented that religion and spirituality play a pivotal role in shaping the health beliefs and behaviours of Muslims, influencing their perception of illness, help-seeking behaviours and overall utilisation of mental health services (Aloud and Rathur, 2009; Padela *et al*., 2012; Bagasra and Mackinem, 2014; Alhomaizi *et al*., 2018). While Muslim Americans may share the same faith, the population itself is very diverse and varying degrees of spirituality further compound its complexity. Muslim Americans differ across ethnicities, socioeconomic status, race, education, degree of religiosity and exposure to life stressors (Padela *et al.*, 2012). This intersectionality of religion and culture makes Muslim mental health a challenging yet rewarding opportunity for researchers to study across the biopsychosocial and public health landscape.

However, barriers to minority engagement in research such as social stigma and cultural mistrust continue to hinder research attempts to understand and address Muslim American mental health needs (Amri and Bemak, 2013; Ciftci *et al*., 2013). For example, many Muslim Americans find themselves unwilling to participate in studies regarding mental illness or substance use for fear of familial and/or community repercussions (Amer and Bagasra, 2013). Others hesitate to engage in mental health research for fear of being misunderstood, misinterpreted or misrepresented by researchers that are not familiar with the complex and nuanced religious beliefs and diverse cultural backgrounds of Muslims Americans (Raiya *et al*., 2007; Amer and Bagasra, 2013). Historically, traditional research designs have been unsuccessful in engaging marginalised communities in the research process and have failed to generate knowledge that meaningfully addresses locally identified problems that reflect the socioeconomic, political and health disparities faced by faith communities and communities of colour (Minkler and Wallerstein, 2003; Becker *et al*., 2005). This has called for social justice-oriented research approaches that go beyond recognising the voices of community members to valuing and utilising their lived experiences in order to successfully create and maintain community health strategies and social change (Jacobson and Rugeley, 2007).

One of the most recognised participatory research approaches is community-based participatory research (CBPR), which has been described as an emancipatory research paradigm that is rooted in social empowerment (Jacobson and Rugeley, 2007). CBPR, as the name suggests, relies on the involvement and subsequent integration of community members who are most affected by the problems during the earliest stages of research design and implementation processes, resulting in the development of prevention programming and interventions that are tailored to each community's specific needs. At its core, CBPR involves engaging the community and one way that has been demonstrated to work successfully in the literature is through the formation of participatory structures such as community advisory boards (CABs) (Newman *et al*., 2011; Ortega *et al*., 2018; Mitchell *et al*., 2020). CABs are usually composed of individuals who represent the community targeted for research and who serve as liaisons between the research team and the communities they serve. In addition to serving as a link, there are several roles of CABs that have been documented in the literature including establishing trust, providing the feedback necessary to provide culturally and religiously sensitive care, recruitment for research studies and assisting in the development, implementation and review of research protocols (Lucero *et al*., 2018).

In this paper, we present a novel case for the formation, operation and evaluation of a community advisory aboard comprised of Muslims residing in the San Francisco Bay Area (SFBA), California that utilised a community based participatory approach to address local Muslim mental health needs. In addition to describing the development of the CAB, the authors present the findings of its evaluation and the synthesis of best processes based on CAB members' feedback.

## Case study background

**Fig. 1.**
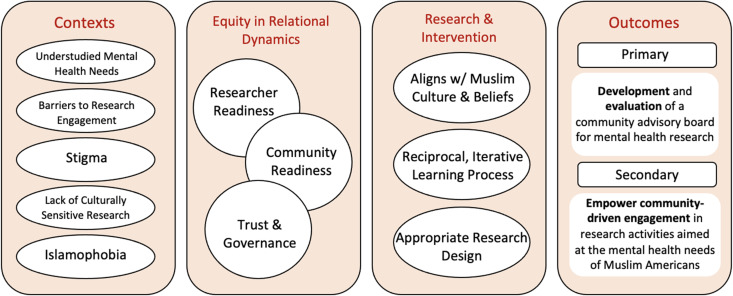
CBPR Conceptual Logic Model – Modified from (Wallerstein and Duran, 2010).

Our approach to the development of the CBPR partnership with Muslim communities in the San Franscisco Bay Area, California was guided by an adaptation of Wallerstein and Duran (2010) that continues to evolve over time (See Fig. 1). Identifying and understanding contextual factors, both epidemiological and sociological that affects a partnership is a key step in the development and sustainability of a partnership.

A summary of the conceptual logic model is illustrated in Fig. 1.

### Study context: characteristics of the partnering population and relational dynamics of the involved institutions

The Stanford Muslim Mental Health Lab & Islamic Psychology (MMHIP) lab was established in 2014 as an academic home for the study of mental health in the context of the Islamic faith and Muslim populations. The Lab aims to provide intellectual resources to clinicians, researchers, trainees, educators, community and religious leaders working with or studying Muslims. In 2016, the Center for Clinical and Translational Research and Education (SPECTRUM) at Stanford University awarded a pilot grant to the MMHIP lab in an effort to enhance an emerging research partnership with the Muslim Community Association (MCA), the largest Muslim community centre in the SFBA. Utilising a CPBR approach, one of the major goals of the project was the establishment of a CAB made up of Muslim representatives residing in SFBA to lead the research partnership and guide the design and implementation of research projects. The primary goal of the CAB was to help the research team design and conduct focus groups to explore barriers and facilitators to utilisation of mental health services among Muslims residing in SFBA.

It is estimated that over 250 000 Muslims reside in the SFBA, California, making it one of the largest Muslim populations in the United States (Senzai and Bazian, 2013). The Muslim community in the SFBA is characterised by its significant ethnic diversity, including but not limited to South Asians, Afghans, Arabs, African Americans, East Asian/Pacific Islanders, White Caucasians and Iranians (Senzai and Bazian, 2013). The diversity of Bay Area Muslims required a partnering Islamic institution that would represent this diversity. We chose the MCA, as it serves a congregation of over 10 000 people. It provides a wide spectrum of services including daily congregational prayers, educational programmes, a full-time Islamic school, a weekend Islamic school, outreach services, social activities, counselling, medical and legal services. Recognising the growing mental health needs of the community, the MCA board members and executive directors have been welcoming to spiritually and culturally sensitive mental health and wellbeing initiatives. This institutional readiness made the MCA an optimal candidate for the partnership.

### Launching of the CAB

Newman *et al*. (2011) have identified three phases that constitute best processes in CAB development: (a) formation, (b) operation and (c) maintenance. In the next section we will highlight the three phases of the Bay Area CAB development.

#### Formation

A purposeful sampling and snowballing design were used to recruit Muslims from the SFBA community. We established a preliminary criterion for recruitment that included any Muslim community member 18 years and older that had lived in the Bay Area for more than one year and could speak and understand English fluently. In coordination with an MCA liaison who is a licensed family and marriage counsellor and was the primary coordinator for MCA-affiliated social and counselling activities at that time. Part of our informed sampling design also involved the input of the PI and co-PI who are part of the MCA community and utilised their social networks and knowledge about the community to identify participants. Through a multi-faced recruitment approach that utilised email and in-person communication strategies, both virtual and in-person meetings were held to discuss the purpose of the CAB and the responsibilities joining it would entail. An initial cohort of fourteen members would go on to become the first SFBA CAB. Once the first cohorts of stakeholders were recruited, they were asked to suggest other community members for recruitment. In total, 24 SFBA community members (eight males and sixteen females) served on the CAB during the year of 2016.

#### Operation

The CAB members agreed to serve a renewable, one-year term and participated in monthly 2-h meetings. The participation was entirely voluntary with no monetary compensation. The primary goal of the CAB was to guide the design and implementation of community sensitive focus groups to explore the barriers and facilitators of their fellow community members' utilisation of mental health services. To help the CAB carry on this role, the research team and the CAB were engaged in collaborative monthly meetings with ongoing mutual learning about the research process and what best fit the community. The CAB was introduced to the principles of CBPR and research process framework which included a brief introduction to the concept of the IRB, ethical research standards and confidentiality, the concept and steps needed to conduct focus groups. Details on the scope of the meetings are included in online Supplementary appendix 1. CAB participation spanned various domains that included providing feedback on the makeup and the needs of the SFBA community. With increasing dedication and motivation of the CAB, members contributed across the entire research design and implementation process, from participating in the development of focus groups manual and formulating the case scenarios that were used to facilitate focus groups discussions to eventually leading the recruitment process, including the development of the recruitment matrix, advertisement of the focus groups and the recruitment of participant inclusion. CAB activities were all conducted during the monthly meetings except for the day of the implementation of the focus groups.

It is important to note that the establishment of the CAB took place in 2016, a year in which a spike of Islamophobia was observed in light of the presidential election cycle (Abu-Ras *et al*., 2018; Lajevardi and Oskooii, 2018) that resulted in heightened levels of distress among Muslims residing in the US. Unsurprisingly, the CAB provided a platform to organise mental health initiatives in order to organically and swiftly respond to changes in the sociopolitical atmosphere. Among these outcomes was the development of a mental health crisis response team for Muslims residing in the SFBA and the development of a searchable directory of Bay Area Muslim Mental Health professionals (Ali and Awaad, 2019). In addition, the CAB took on the responsibility of creating a research agenda to guide future research projects.

#### Maintenance and sustainability

Productive CAB partnerships are encouraged to last beyond the life of their research project (Blumenthal, 2006), and evaluation is essential to that goal. Evaluation provides the opportunity for partners to identify problems and plan for solutions that strengthen the group and improve overall performance, as well as provide member satisfaction (Newman *et al*., 2011).

## Methods

To obtain an independent, unbiased, assessment of the partnership, an evaluation was conducted by a team from the Office of Community Engagement, Center for Population Health Sciences at Stanford University. The evaluation team had previous knowledge about the project but was not involved in the development or maintenance of the partnership. The evaluation team consisted of two evaluators who had experience in assessing community partnerships and evaluating CABs. The goal of the evaluation was to use mixed method data collection strategies. The evaluation process was launched six months after the first CAB inception meeting. The evaluation was conducted over three meetings in which the evaluation team met with the CAB in a group interview format in the absence of the Stanford University research team. The first evaluation meeting included introduction to the goal of the evaluation and eliciting feedback from the CAB on the evaluation process. The second meeting included an in-person evaluation during which the evaluation team collected qualitative and quantitative data using anonymous individual surveys and small group open discussions. The questions used in the data collection process were based on an instrument for evaluating dimensions of group dynamics within CBPR partnerships (Schulz *et al.*, 2003). Details of the survey questions are included in the online Supplementary appendix. The third meeting goal was to present the results of the evaluation to the CAB members. Fourteen CAB members participated in the evaluation session. Eight of the fourteen members had been involved since inception of the group.

### Data analysis

Our research team conducted a thematic analysis of the qualitative data provided by the evaluation team. Thematic analysis of individual questionnaire responses and open coding of transcripts provided by the evaluation team of the open-form discussion was conducted using Newman *et al*.'s framework which conceptualises the success of a CAB using three main strategies that focus on formation, operation and maintenance (2011). Formation processes address key activities related to defining the role and purpose of a CAB and subsequent identification and recruitment of key stakeholders from the community for participation in the CAB. Operation processes address the development of procedures to guide the logistical operation of the CAB, the development of guiding principles to assure the values of the community are represented and respected, and the establishment of leadership and decision-making protocols. Maintenance processes address the evaluation of a CAB's actions and outcomes and plans for sustainability. Ongoing attention to evaluation and sustainability is essential to the maintenance of both newly formed and long-standing CABs.

## Results

### Evaluation of the Stanford-SFBA partnership: CAB formation

Three main topics emerged from the analysis of the CAB members' feedback regarding the formation process: recruitment diversity, challenges in recruitment and refinement of membership. The collective responses indicated that most CAB members were satisfied with the diversity represented in the members of the board. The CAB expressed interest in more focused recruiting of the following professional, ethnic and religious representations: religious leaders and imams, men, board members of various masjids within the community, Shia Muslims – a minority Muslim sect, elders, youth, Bengalis and African Americans.

One of the participants commented on increasing the diversity of recruitment saying, ‘I really want to see more representation for the African-American community and also pockets of immigrant communities.’ Another participant shared that ‘[they] think that we are tapping into certain groups that are more religiously affiliated. Perhaps research on non-religious spaces where Muslims get support would be helpful.’ As for recruitment of religious leaders' another participant stated: ‘Perhaps incorporate more Imams (which I know is a goal) and more men.’ Suggested strategies to increase diversity were: (1) Physically rotating the site of meeting to create accessibility for all (2) Announcements at centres where marginalised communities congregate using social media and creating third spaces (3) Encouraging current CAB members to recruit individuals who are not represented.

### Evaluation of the Stanford-SFBA partnership: CAB operation

When presented with a multiple-choice question developed by the evaluation team to elicit understanding of the purpose of the group as perceived by community members, the consensus amongst participants regarding applicable statements amongst those provided were as follows: to assess, recommend and evaluate the availability and accessibility of mental health services to the Muslim community (43%), to evaluate and analyse the mental health care needs of the Muslim community (29%), to understand mental health services barriers/stigma (14%), to raise awareness of mental illness within the Bay Area Muslim community (7%) and to gather a group of educated Muslim professionals to get together and discuss the matter going on in their worlds (7%). Quantitative assessment of group dynamics also indicated overall satisfaction with the process of running the CAB. 86% of participants found the group meeting useful, 79% reported that they enjoyed attending the group meetings, and 71% reported that the meetings were well organised.

In addition to overall group satisfaction and understanding, two main themes were discussed amongst participants during the open-forum discussion regarding CAB operation: successful strategies of CAB operation and recommendations for further enhancements. Successful strategies were identified based on expressed satisfaction from CAB members regarding the following topics: leadership, the positive culture that prevailed, the group dynamics, meeting workflows and direct participation and engagement in research design and implementation (see Table 1).

**Table 1.** Example quotes on successful strategies for CAB operation

Leadership	‘The group leadership has done an excellent job of directing us with focus and task without overburdening the general members.’
Group Dynamics	‘The meetings are useful, positive, filled with trust.’ ‘Knowledgeable, competent group…’ ‘Time to socialize and get to know one another was valuable.’
Meeting logistics	‘Always have an agenda, quick summary with tasks…’
Research Engagement and Participation	‘My favorite part of the entire meeting has been actually physically running the groups. Working directly with the youth has been fascinating for me as our community is well informed and actually really open to expressing their feelings.’ ‘It was a good experience and fruitful to engage our community in a deep and structured way. I was not directly involved but was onsite to help that day. Seemed very smooth going’

Recommendations for operation enhancement included further refinement of logistical operations with sustainability in mind, iterative development of CAB training and educational curriculum and the possibility of compensation of CAB members for their participation. One member suggested future utilisation of strategies to create opportunities to enhance creativity and heterogeneity of CAB feedback. A few CAB members also reported interest in incorporating spirituality into meetings stating that ‘[they] would like to see a spiritual warm up or prayer scheduled into the meeting.’

### Evaluation of Stanford-MCA partnership: CAB maintenance

The CAB members expressed interest in maintaining the work beyond the focus groups and crafting short and long-term outcomes. The goals suggested included more research activities, mental health interventions, building collaborations and mental health advocacy. One participant expressed that he would like the goals to include, ‘raising mental health awareness, eliminating stigma, and easier access to services and providers.’ Another member advocated for the development of more ‘support groups and discussions [for adults, young adults, and youth], focus groups, and more meetings with key stakeholders in our community.’ Some participants stressed the importance of building collaborations with other Muslim and faith-based mental health organisations. One participant offered insight into the nature of these collaborations and the types of institutions that should be involved, ‘think tanks, meetings, and continuous dialogue to identify other mental health or social welfare organizations, particularly faith-based organizations; and collaborate, exchange ideas, and work on projects, all of which should start at the county level.’

## Discussion

This case study reviewed the formation, operation and maintenance of a CAB using best processes. The formation and operation of the CAB was founded on CBPR principles that included a strength-based approach that leverages existing resources and networks from within the community, models respect and values mutual learning and power sharing. The long-standing relationship between the Stanford Muslim Mental Health and Islamic Psychology Lab and the MCA provided an invaluable opportunity to build upon this tenet of CBPR by providing a strong foundation for the formation of the Stanford-SFBA CAB. Identifying key stakeholders who have credibility in the community is a vital step in building trust with a partnering community in any CBPR model. In our case, the MCA was the trustworthy entity that facilitated the inception of the partnership and provided a safe physical space rooted in shared values and morals that allowed for the partnership to flourish. In addition, the research team's lead investigator is from the Bay Area Muslim community and was well-known and respected for both her scientific and religious backgrounds, both of which provided credibility and comfort to research participants that this project would be spiritually and culturally congruent.

As part of the maintenance phase (Newman *et al*., 2011), an evaluation was performed that was successful in providing insight into the design and implementation of the CAB with the goal of promoting sustainability beyond the initial research initiative. Some of the strengths highlighted by CAB members centred around (a) group satisfaction, (b) perceived roles and responsibilities and (c) research engagement. CAB members found the greatest satisfaction out of their contributions through direct participation in the research activities that were conducted by the university-CAB team. The evaluation also revealed that the prioritisation of mutual learning, respect, capacity building and co-leadership structures may contribute to the maintenance and sustainability of CABs that extend beyond the scope of the community-research partnership. This was achieved by supporting the operation of the CAB using an iterative design. This allowed the CAB to carry on its primary goal which is to guide the implementation of focus groups and be flexible enough to accommodate evolving community needs at the time. This iterative design resulted in the creation of a list of research initiatives by the CAB to inform community interventions in the SFBA Muslim communities. The list is currently being used to direct research and mental health initiatives in the SFBA and included the establishment of community listening meetings, an initiative aimed at obtaining a wider assessment of the mental health needs of the SFBA Muslim communities.

### Challenges

Although feedback from CAB members and its evaluation was largely positive, key challenges were highlighted that were largely related to recruitment diversity, retention strategies and increased support of CAB member participation through monetary compensations. Given that the SFBA is the home to very diverse Muslim communities, challenges in recruiting representatives that account for all possible self-identifying groups was expected. However, through an iterative and social-justice integrated recruitment paradigm, CAB members are already improving community representation through continuous recruitment of underrepresented ethnicities, religious sectors and organisations. Retention continues to be a challenge to maintain a CAB with active participation of 8–14 diverse community members in meetings. Although the Stanford Muslim Mental Health and Islamic Psychology Lab team continues to apply for grants to secure monetary compensations, evaluation of CAB feedback only further highlights another of the five key tenets of CBPR according to McAllister *et al*. (2003): sufficient support for the community partners. Given the limitations of funding, and pre-existing awareness of such limitations in research aimed at addressing health disparities through a CBPR-framework, our CAB evaluation further emphasises the need for additional avenues of financial support (Killawi *et al*., 2015).

## Conclusion

The Stanford-SFBA partnership, which was guided by a commitment to community-rooted research, brought religious and cultural sensitivity and contributed towards meaningful translational research in the field of Muslim mental health. The development of the Stanford-SFBA CAB and its subsequent outcomes, both intended and unintended, have exceeded our expectations as a research team. While our primary aim was to include community voices in the implementation of religiously and culturally sensitive focus groups, the CAB was empowered to become a platform for the inception of many mental health projects. Despite the need for public health funding, the CAB continues to function at a high momentum, fuelled by self-efficacy, dedication of its members and support from the SFBA Muslim communities.

## Data Availability

The data that support the findings of this study are available from the corresponding author upon reasonable request.

## Supplementary material

For supplementary material accompanying this paper visit http://doi.org/10.1017/S2045796022000786. click here to view supplementary material
