# Supplementary material for: The Bay Area Muslim mental health community advisory board: evaluation of a community based participatory approach
Source: Epidemiol Psychiatr Sci. 2023 Jan 31;32:e7. doi: 10.1017/S2045796022000786 (PMC9971854; doi:10.1017/S2045796022000786)
Supplement: Supplementary file 1 [file S2045796022000786sup001.docx]

Appendix 1

| Meeting number | Outcomes |
| --- | --- |
| 1 | Introductions of researchers and community members.  Discussion of grant goals.  Community members shared why they are interested in participation.  Conducted brain-storming exercise on barriers to seeking out mental health care in the Muslim community. |
| 2 | Introductions for new members.  CAB brainstorming meeting norms and sharing ideas on how to conduct effective meetings.  Examining expectations and documenting CAB roles.  Explanation of the concept of CBPR and discussing different models.  Introducing the concept of IRB and discussing confidentiality.  Introducing the idea of the focus groups and discussing its appropriateness to the community.  CAB developing recruitment matrix for the focus groups.. |
| 3 | Discussing locations and logistics of the focus groups.  CAB nominates community members for recruitment and discussing their fit to the recruitment matrix.  CAB developed a recruitment plan and led the recruitment process.  CAB creates a list of mental health needs and research topics that are important to Bay Area Muslims. |
| 4 | Assigning roles for the CAB (bringing snacks, focus groups registration, audiotaping, facilitation).  Presenting the focus Group facilitation training for the CAB.  CAB developing case scenarios for the focus groups with the help of the PI. |
| 5 | Conducting four focus groups.  Post focus group debrief. |
| 6 | Discussing next steps.  Finding funding  Inception of the Crisis Response team. |
| 7 | Evaluation of the partnership by a team from the Office of Community Engagement at the Center for Population Health Sciences at Stanford. |

Appendix 2:

Selected questions from (Schulz, Israel, & Lantz, 2002) that were utilized in the evaluation process:

| **CAB Evaluation Questions** |
| --- |
| What is your understanding of the purpose of this group? |
| Thoughts regarding meeting…   - 1. Usefulness   2. Enjoyment   3. Organization   4. Frequency   5. Agenda   6. Relational Dynamics   7. Location |
| How satisfied are you with the way group meetings are prepared and structured? What  would you do differently? |
| How comfortable are you about expressing your opinion in group meetings? |
| Since you joined the group, would you say group members’ are more willing to express  their points of view, the same, or less willing to express their opinions? |
| In your opinion, how much trust and openness exists between group members? |
| What was your experience in forming and/or conducting the focus groups? |
| How important do you think the work of the group is to the community as a whole? |
| It is our intent in these group meetings to be respectful to the Muslim culture. How can  the meetings be more respectful to the Muslim Culture? |
| Do we have enough diversity on the CAB? If not, what steps should we take to become  more diverse? |
| How long do you intend to be a member of this CAB? |
| Moving forward, what do you see as the vision for this group? |
| What ideas do you have for sustaining the group? |
| 14. What ideas do you have for activities organized by the group? |
| 15. What ideas do you have for connecting with other groups doing similar work? |

| **Small Group Discussion** |
| --- |
| Count off in 3’s |
| Groups: |
| Group 1: How to ensure that the environment is safe for all diverse opinions? |
| Group 2: How to enhance diversity and inclusion? |
| Group 3: Plan for sustainability |
| Brainstorm ideas for CAB consideration |
| Pick top 3-5 ideas to share |
| Report out |
